# Comprehensive Analysis of LncRNA Reveals the Temporal-Specific Module of Goat Skeletal Muscle Development

**DOI:** 10.3390/ijms20163950

**Published:** 2019-08-14

**Authors:** Yinghui Ling, Qi Zheng, Menghua Sui, Lu Zhu, Lina Xu, Yunhai Zhang, Ya Liu, Fugui Fang, Mingxing Chu, Yuehui Ma, Xiaorong Zhang

**Affiliations:** 1 College of Animal Science and Technology, Anhui Agricultural University, Hefei 230036, China; 2 School of Natural and Environmental Sciences, Newcastle University, Newcastle upon Tyne NE1 7RU, UK; 3 Local Animal Genetic Resources Conservation and Biobreeding Laboratory of Anhui Province, Hefei 230031, China; 4 Institute of Plant Protection and Agro-Products Safety, Anhui Academy of Agricultural Sciences, Hefei 230036, China; 5 Key Laboratory of Farm Animal Genetic Resources and Germplasm Innovation of Ministry of Agriculture, Chinese Academy of Agricultural Sciences, Beijing 100193, China

**Keywords:** lncRNA, skeletal muscle, transcriptome, goat, development

## Abstract

A series of complex processes regulate muscle development, and lncRNAs play essential roles in the regulation of skeletal myogenesis. Using RNA sequencing, we profiled the lncRNA expression during goat (*Capra hircus*) skeletal muscle development, which included seven stages across fetal 45 (F45), 65 (F65), 90 (F90), 120 (F120), 135 (F135) days, born for 24 h (B1) and 90 (B90) days. A total of 15,079 lncRNAs were identified in the seven stages, and they were less conservative with other species (human, cow, and mouse). Among them, 547 were differentially expressed, and they divided the seven stages into three functional transition periods. Following weighted gene co-expression network analysis (WGCNA), five lncRNA modules specific for developmental stages were defined as three types: ‘Early modules’, ‘late modules’, and ‘individual-stage-specific modules’. The enrichment content showed that ‘early modules’ were related to muscle structure formation, ‘late modules’ participated in the ‘p53 signaling pathway’ and other pathways, the F90-highly related module was involved in the ‘MAPK signaling pathway’, and other pathways. Furthermore, we identified hub-lncRNA in three types of modules, and LNC_011371, LNC_ 007561, and LNC_001728 may play important roles in goat skeletal muscle. These data will facilitate further exploration of skeletal muscle lncRNA functions at different developmental stages in goats.

## 1. Introduction

Most non-protein coding regions of the genome have historically been regarded as ′junk DNA′. However, the rapid development of high-throughput sequencing technologies over the past decade has led to intensive research on the non-coding parts of the genome. Although less than 2% of mammalian genomes encode proteins, most of the nucleotides in the genome are detectably transcribed under certain conditions [1]. Long non-coding RNA (lncRNA) is contained in a large number of non-protein encoded transcripts and is defined as a transcript of greater than 200 nt in length with a 5-terminal cap structure and a 3-terminal polyA tail and is primarily transcribed by RNA polymerase II [2]. The number of identified lncRNAs is close to that of the protein-encoding mRNAs (GENCODE V25, http://www.gencodegenes.org/). Although most lncRNAs are poorly conserved and their expression levels are significantly below that of mRNA, they show an interesting cell-type-specific expression pattern [3,4]. In addition, a huge number of lncRNAs have been found to have an indispensable role in development, including the regulation of cell fate decisions [5], cell differentiation [6,7], cell cycling and proliferation [8,9], and apoptosis and aging [10,11].

Domestic goats (*Capra hircus*) are among the most essential commercially farmed animals that have attracted increasing attention as a viable source of meat production, which is closely related to fetal muscle development. Secondary (fetal) myogenesis relies on the fusion of fetal progenitor cells which produce secondary muscle fibers with no net increase in the number of muscle fibers after birth [12,13]. Hence, it is crucial to elucidate the changes in skeletal muscle formation and the underlying molecular mechanisms during prenatal and postnatal development. Numerous studies to date have shown that lncRNAs regulate cell biological processes in skeletal muscle through a variety of gene regulatory mechanisms. Examples of these functions include lncIRS1, linc-MD1, linc-RAM, and others [14,15,16]. Although researchers have explored the types of skeletal muscle lncRNAs expressed at different stages of goat development, these prior studies have only explored the potential role of these molecules. The exact mechanism by which lncRNAs play a role in the complex development of skeletal muscle thus remains unclear.

In this present study, we performed RNA-seq analysis on goat skeletal muscles at 5 fetal stages, in which the ewe has been pregnant for 45 d (F45), 65 d (F65), 90 d (F90), 120 d (F120), and 135 d (F135), and 2 kid stages, in which the kids were born for 24 h (B1) and 90 days (B90) postnatally. LncRNAs were systematically identified from the data, and structural features, sequence conservation, temporal expression patterns and potential functions were elucidated. The findings from these analyses will be of great use in future explorations of skeletal muscle lncRNA functions and mechanisms at different stages of development.

## 2. Results

### 2.1. Identification and Characteristics of the Goat Skeletal Muscle Transcriptome

**Figure 1:**
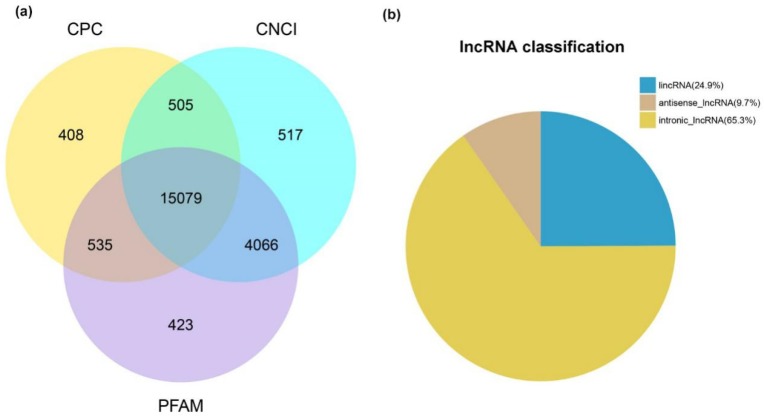
Identification of goat skeletal muscle long non-coding RNAs (lncRNAs). (**a**) Venn diagram presentation for prediction of coding potential using three software identifications, including CPC analysis, CNCI analysis, and Pfam protein domain analysis. (**b**) Pie chart of different types of lncRNAs. Blue: lincRNA; Khaki: antisense_lncRNA; Yellow: intronic_lncRNA.

To comprehensively profile the goat skeletal muscle transcriptome, libraries of 21 *longissimus dorsi* muscle samples at seven development stages (F45, F65, F90, F120, F135, B1 and B90; each stage contained three repetitions) were generated. In each RNA-seq data set, the average sample sequencing depth of the lncRNAs was 113.04 million raw reads per sample (Table S1). After discarding adaptor sequences and low-quality reads, 109.06 million clean reads were obtained per sample of the lncRNA sequencing libraries (Table S1). Next, we found 103.63 million reads were mapped to the reference sequence using HISAT2, and 91.89∼96.55% of the known annotated genes could be detected and characterized in the lncRNA sequencing libraries (Table S1). To identify lncRNAs from different goat development stages, the transcripts were assembled and reconstructed into a total of 400,483 transcripts using StringTie software [17]. Ultimately, 15,079 multi-exonic lncRNAs, located on 228 genomic loci, were identified from the remaining transcripts, among which 1466 (9.70%) originated from anti-sense regions (Figure 1 and Table S2). 

### 2.2. Characteristic and Conservation Analysis of Goat LncRNA

**Figure 2:**
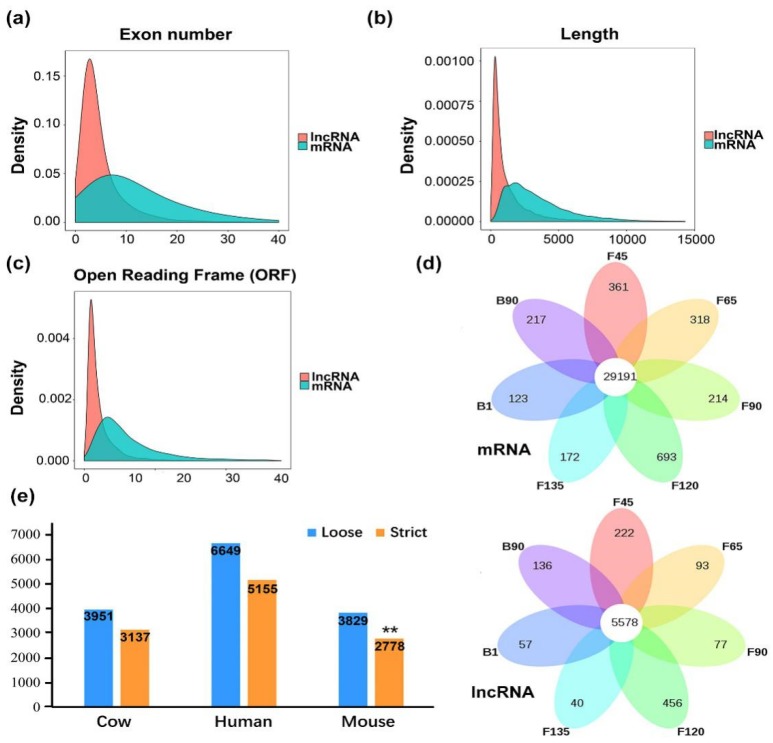
Goat skeletal muscle structure and conservation. (**a**–**c**) Exon number, transcript length and open reading frame (ORF) length distribution of transcripts for all lncRNAs and mRNAs in goat skeletal muscle. Red: lncRNA; Blue: mRNA. (**d**) Venn diagram of detected mRNAs (top) and lncRNA (bottom) at seven stages. (**e**) The number of NONCODE lncRNAs in cow, human, and mouse that are either a loose (E-value < 1 × 10^−3^) or strict (E-value < 1 × 10^−10^) threshold by BLASTN. (**) *p*-value < 0.05 by Fisher’s exact test with cow used as the background.

It is important to maximize our understanding of lncRNAs and mRNAs through a combination of multiple structural features. LncRNAs tend to contain fewer exons than protein-coding transcripts, and they are shorter than mRNAs in length due to fewer exons (Figure 2a,b). In addition, lncRNAs in the datasets also had a shorter ORF than the mRNAs (Figure 2c). In all stages, 68% of all mRNAs (from total) were co-expressed, whereas only 37% of lncRNAs (5578 from total) were co-expressed among the seven stages. These data indicated that lncRNA expression in skeletal muscle is less conserved than that of mRNA (Figure 2d).

To further explore the conservation of *longissimus dorsi* muscle lncRNAs, specific lncRNAs from human (172,216), cow (23,515), and mouse (131,697), downloaded from the NONCODE database, were used for comparative analysis. The analysis revealed that 15,079 goat muscle lncRNAs were aligned to 6649 (44.09%) lncRNAs from human, 3951 (26.20%) from cow, and 3829 (25.39%) from mouse after BLAST filtering. It was interesting to note that the homologs identified among the goat muscle lncRNAs were not significantly reduced by increasing the BLAST stringency compared with cow and human, whereas those of goat and mouse were significantly (*p* < 0.05) decreased (Figure 2e). This confirmed the low conservation between lncRNAs of different species, but the similarity of lncRNA between goats and humans was higher.

Because human lncRNAs showed a higher degree of alignment with those from goat, phastCons software was used to assess the conservation scores for known human lncRNAs from which 418 with scores above 0.8 were selected for alignment with goat lncRNAs in the datasets (Table S3). When lncRNAs with a conservation score > 0.8 were subjected to BLAST analysis with goat skeletal muscle lncRNAs, conservation scores were obtained for 111 molecules (26.55% of identified human lncRNAs). After reverse alignment, only 132 (31.57% of identified human lncRNAs) lncRNAs achieved conservation scores (Table S4).

### 2.3. Dynamic Expression of LncRNA and mRNA

**Figure 3:**
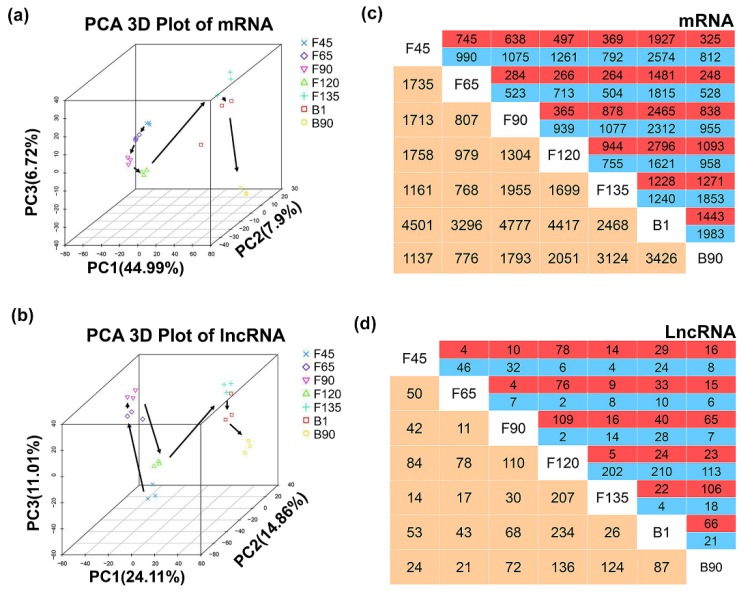
Expression patterns of lncRNAs and mRNAs during goat skeletal muscle development. (**a**,**b**) Principal component analysis (PCA) analysis of lncRNAs and mRNAs in 21 samples of goat skeletal muscle at seven different stages of development. The same color represents the same stage. The arrows indicate the direction of development between successive muscle stages. (**c**,**d**) Number of differentially expressed mRNAs (top) and lncRNAs (bottom) showing up- (red) or down- (blue) regulation during development. Yellow: number of total differential genes between two stages.

Principal component analysis (PCA) was performed to better understand the temporal expression pattern of lncRNAs and mRNAs obtained from the datasets. The data for three samples at the same stage were clustered together and the developmental order was also accurately captured from F45 to B90 (Figure 3a,b). Expression patterns of mRNA were similar at F45, F65, and F90, whereas major changes had taken place in the continuous development stages between F120 and F135, except for one B1 sample of mRNAs (Figure 3a). Furthermore, the lncRNA expression patterns were similar from F65 to F90 and F135 to B1, with the other stages clustered separately (Figure 3b).

Next, a total of 547 lncRNAs and 12,611 mRNAs were differentially expressed (*p* < 0.05) during the seven stages (Tables S5 and S6). All of the differentially expressed mRNAs (DEmRNAs) showed a consistent pattern with PCA (Figure S1a). And Similar to PCA, a heat map of differentially expressed lncRNAs (DElncRNAs) also showed that all DElncRNAs had the most similar expression patterns from F65 to F90, and from F135 to B1 (Figure S1b). This finding indicated that the birth process does not affect the regulation of lncRNA during skeletal muscle development. In the two adjacent age groups, the differences in the DEmRNAs occurred from B1 to B90, and the smallest changes occurred from F65 to F90 (Figure 3b). DElncRNAs showed the greatest change from F120 to F135, involving 207 DElncRNAs, while only 11 lncRNAs showed changes from F65 to F90 (Figure 3d). Interestingly, in any two stages, DEmRNAs at B1 had changed the most from other stages except F135, and DElncRNAs at F120 showed the greatest difference from the other stages (Figure 3c,d). These data divide the stages from F45 to B90 into three skeletal muscle development stages—(1) F45 to F90; (2) F135 to B90; and (3) the transition period mainly occurs in F120. 

### 2.4. Temporal Expression Patterns of LncRNAs

**Figure 4:**
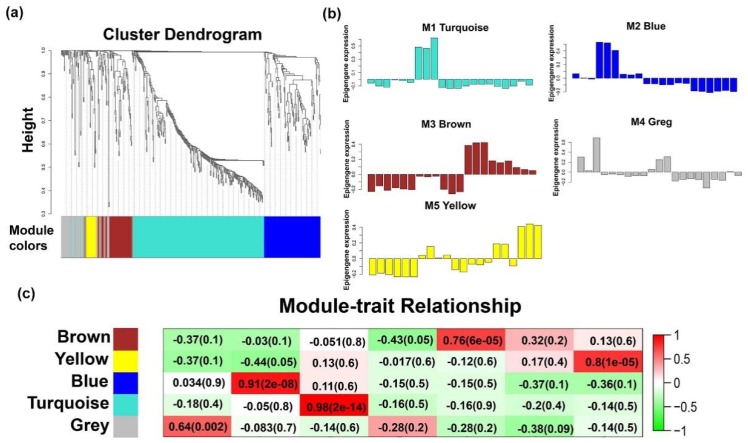
Expression modules of lncRNAs determined by weighted gene co-expression network analysis (WGCNA). (**a**) Hierarchical cluster tree of all differentially expressed lncRNA modules. Modules are corresponded to the branch and are marked by a color, such as color strips under the tree. (**b**) Hierarchical clustering heat map of all differentially expressed lncRNAs by sample. (**c**) Eigengene bar plot of all modules of lncRNAs. The colors correspond to the individual lncRNA modules. The order of the samples is F45, F65, F90, F120, F135, B1, and B90. Each developmental stage contains three biological samples.

To demonstrate the dynamics of lncRNAs at different stages of goat skeletal muscle development, the expression patterns of DElncRNAs (547) were clustered using weighted gene co-expression network analysis (WGCNA). A total of 5 lncRNA transcriptional modules (M1–5) were identified in goat skeletal muscle with all the modules showing a strong correlation (correlation > 0.6, *p*-value < 0.05) with specific developmental stages (Figure 4a,b). To further explore the lncRNAs in each specific module, we described the modules using eigengene value graphing (i.e., by ′color′ corresponding to a cluster dendrogram) (Figure 4c). We focused on modules that were highly correlated with developmental stages and defined these temporal-specific modules as three groups: Early modules, late modules, and individual-stage-specific modules.

#### 2.4.1. LncRNAs Regulate Muscle Formation During Early Development

**Figure 5:**
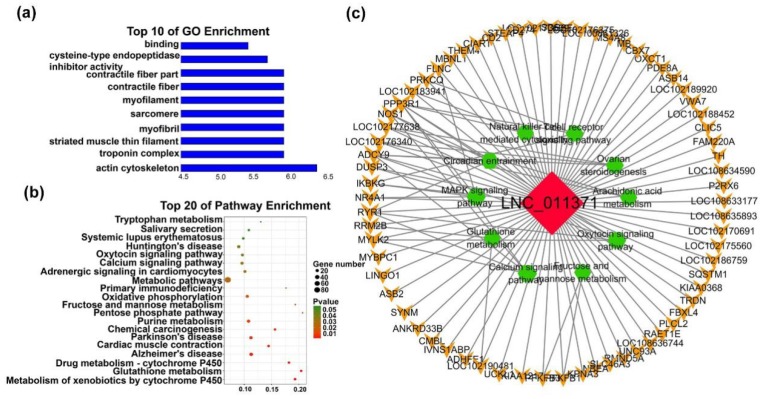
Functional and pathway enrichment of the early module. (**a**) Bar plots showing the top 10 enriched GO terms in M2. The length of the bars indicates significance (−log_10_ *P*-value). (**b**) Top 20 enriched gene pathway terms of the all M2 lncRNAs. (**c**) Function network of the LNC_011371. The green hexagonal denotes the pathway analysis, the red diamond represents lncRNA, orange V shape represents mRNAs, and the size is expressed in degrees.

The characteristics of early transcriptional modules are mainly related to the early stages of development, which are unrelated to the late stage of development, including M2 (blue, 279 lncRNAs) for the lncRNA modules (Figure 5c). The main positive correlations for the early lncRNA module was from F45 to F90. Some mRNAs were bound directly by M2 lncRNAs, such as *JAK3*, *MEF2 A*, myosin heavy chain families (*MYH13*, *MYH14,* and *MYH8*), and Troponin T families (*TNNT1* and *TNNT2*), which are associated with muscle proliferation, differentiation, structural formation, and muscle fiber types (Table 1). Notably, nine of the top 10 GO terms were enriched in muscle fiber structures, such as ‘actin cytoskeleton’, ‘troponin complex’, ‘striated muscle thin filament’ and others, which indicated that the host genes of ‘early lncRNAs’ are associated with muscle structure formation (Figure 5a, Table S7). Additionally, M2 lncRNAs were enriched in ‘calcium signaling pathway’, ‘cardiac muscle contraction’ and other patuways that association with muscle (Figure 5b, Table S8). Then, we focused on lncRNAs with |fold-change (FC)| > 2, *p*-value < 0.05. Subnetwork hub-lncRNAs (degree > 10) shown in the figure (Figure S2). LNC_011371, the top degree of the network, also target the muscle-related mRNAs, which indicates that LNC_011371 functions to maintain normal muscle development and promote proliferation or differentiation in the early stages of skeletal development (Figure 5c).

#### 2.4.2. LncRNAs in Late Modules Are Associated With Muscle Differentiation and Hypertrophy

**Figure 6:**
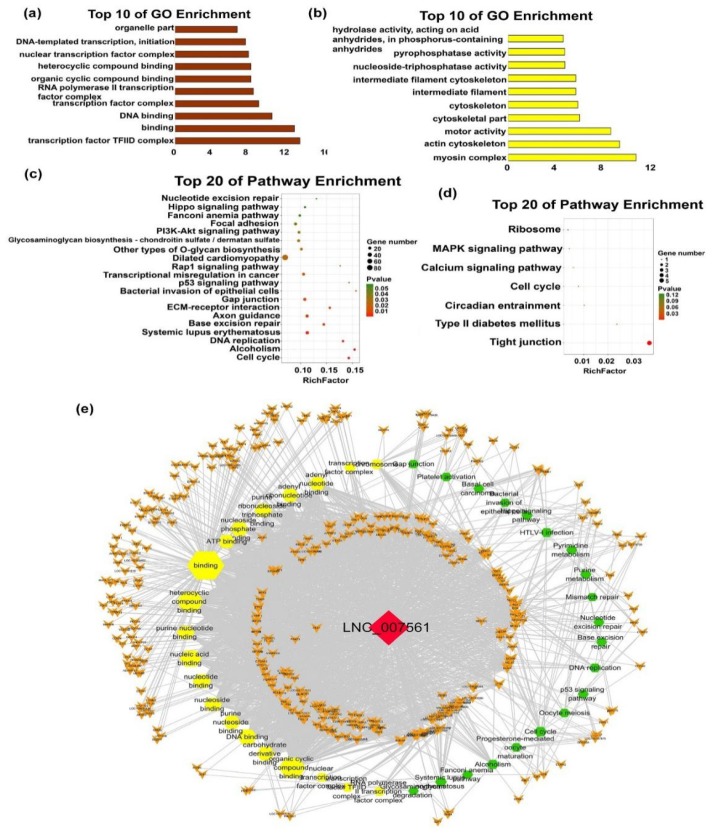
Functional and pathway enrichment of late modules. (**a**,**b**) Bar plots showing the top 10 enriched GO terms in M3 (Brown) and M5 (Yellow). The length of the bars indicates significance (−log_10_ *P*-value). (**c**,**d**) Top 20 enriched gene pathway terms of the all M3 (**c**) and M5 (**d**) lncRNAs. (**e**) Function network of the LNC_007561. The green hexagonal denotes the pathway analysis, yellow hexagonal denotes enrichen GO terms, the red diamond represents lncRNA, the orange V shape represents mRNAs, and the size is expressed in degrees.

Among the lncRNA modules, M3 (brown, 122 lncRNAs) showed a continuously positive correlation with F135 to B90, with 60 of the molecules in this module targeting 1650 genes (Figure 5c). The target genes of M3 lncRNAs involved in muscle differentiation and hypertrophy genes, such as *TCF4*, *EYA2*, and others, are listed in Table 1. Functional analysis of M3 lncRNAs revealed that the targeted genes are enriched in 84 GO terms which may mainly be related to ‘transcription factor complex’, ‘regulation of cell cycle’ and others, and that the GO terms of the lncRNAs in M5 are related to the growth of muscle fibers (Figure 6a,b; Table S7). Pathway analysis further showed that the M3 lncRNAs were mainly enriched for terms connect to development and processing of genetic information and the M5 lncRNAs were only enriched for ‘tight junctions’, which may associate with the shape of muscle fibers (Figure 6c,d; Table S8). Next, we further explored the rigorously screened (|FC| > 2, *p*-value < 0.05) lncRNA-mRNA network of M3 (Figure S3). The subnetwork of M3 hub-lncRNAs which contains nine late lncRNAs are shown in Figure S4. Notably, LNC_007561_targets 560 genes which are enriched in 105 GO terms and 20 pathways, including both ‘cell cycle’, ‘DNA replication’, and others (Figure 6e).

#### 2.4.3. Functions of Individual-Stage-Specific LncRNAs

**Figure 7:**
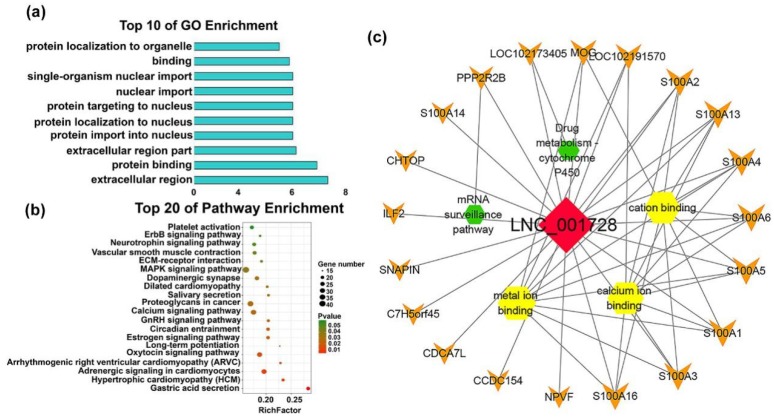
Functional and pathway enrichment of module 1. (**a**) Bar plots showing the top 10 enriched GO terms in M1. The length of the bars indicates significance (−log_10_ *P*-value). (**b**) Top 20 enriched gene pathway terms of the all M1 lncRNAs. (**c**) Function network of the LNC_001728. The green hexagonal denotes the pathway analysis, yellow hexagonal denotes enriched GO terms, red diamond represents lncRNA, orange V shape represents mRNAs, and the size is expressed in degrees.

M1 (Turquoise) was the largest lncRNA module, containing 279 lncRNAs and was the only module to be related (correlation = 0.98, *p*-value = 2×10^−14^) to F90, which was a strong association. Functional analysis of M1, the target genes of lncRNAs, were found to be mainly related to the locational migration of proteins, such as ‘protein import into nucleus’, ‘protein targeting to nucleus’, ‘protein targeting to nucleus’ and others, which revealed that the lncRNAs active at F90 help proteins transfer to the nucleus and activate cells (Figure 7a, Table S7). Notably, these lncRNAs were found to be enriched in some interesting pathways, such as the ‘circadian entrainment pathway’ that is relevant to myogenesis and metabolism [36]. Additionally, changes in the ‘oxytocin signaling pathway’ and ‘MAPK signaling pathway’ regulate the regeneration and proliferation of muscle stem cells (Figure 7b, Table S8) [37]. The lncRNA-mRNA hub-subnetwork is shown in Figure S4 and the node with degree > 10 can be observed. LNC_001728, which has the top degree (20) of the network, also targets the muscle-related mRNAs, such as AS100 families, *PPP2R2 B*, *MOG*, and others (Figure 7c). Furthermore, the lncRNAs of module 4 (M4, grey, 62 lncRNAs) are associate with F135 and F45, but were mainly high correlated with F45 (correlation = 0.64, *p*-value = 0.002). Although lncRNAs in M4 were not enriched in GO terms, they targeted *MARK2*, *MEF2 C*, *MYC* and other genes that are associated with muscle growth (Table 1).

### 2.5. RT-qPCR Validation of DElncRNAs

**Figure 8:**
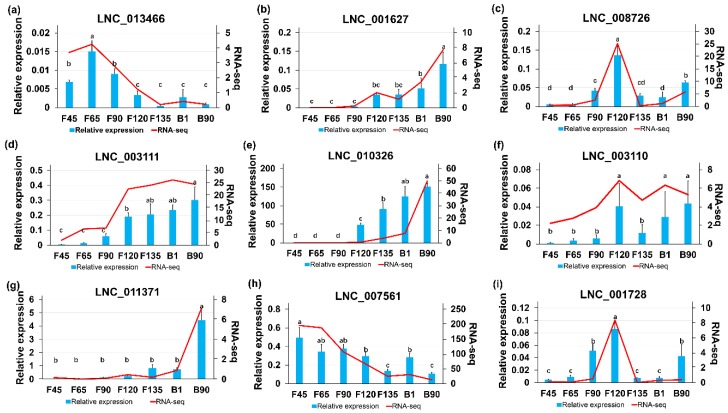
Validation of six differentially expressed lncRNAs by qPCR and RNA-seq. (**a**–**i**) qPCR (bar chart, blue) and RNA-seq expression (line chart, red) validation of the indicated lncRNAs in modules. The letters on the column diagram indicate significant (*p* < 0.05) differences at different stages.

To validate data from RNA sequencing, the expression levels of 6 lncRNAs were randomly selected from DElncRNAs and examined by RT-qPCR at the seven developmental stages under analysis. The results showed that the expression of 6 lncRNAs was consistent with the expression trends calculated from the RNA-seq data (Figure 8a–f). In addition, the three hub-lncRNAs (LNC_011371, LNC_007561 and LNC_001728) screened in Module 1, Module 2, and Module 3 were also verified by RT-qPCR. Their expression levels also had a consistent trend with RNA-seq data (Figure 8g–i).

## 3. Discussion

Transcriptomic data from different species of skeletal muscle have gradually been produced and analyzed. Terry et al. revealed the mRNA transcriptional diversity of mouse and rat skeletal muscle, smooth muscle, and myocardial tissue, and identified candidate genes that may drive tissue specialization [38]. Moreover, 6924 DElncRNAs were obtained from the fetus, lamb, and adult sheep skeletal muscle, and the expression profiles of seven DElncRNAs and their target genes were investigated as to which played vital roles in muscle growth [39]. In addition, the researchers have characterized the lncRNA expression profiles of goat skeletal muscle in four stages. They identified 577 DElncRNAs and explored their *cis* and *trans* effects [40]. In our present study, we analyzed the expression profile of lncRNAs during the seven stages of skeletal muscle development before and after goat birth, characterized the temporal expression patterns of differentially expressed lncRNAs, and displayed the lncRNA–mRNA interaction network of each temporal pattern. These data provide new insights into the role of lncRNA in the evolution of muscle development at different stages and enrich the existing lncRNA resources of mammalian skeletal muscle.

It is essential to maximize our understanding of lncRNAs and mRNAs through a combination of multiple structural features. Consistent with other research findings, our current data indicate that lncRNAs have fewer exons, shorter lengths, and shorter ORFs than mRNA [41]. Besides, the conservation of lncRNAs at the different stages of skeletal muscle development on goat was lower than that of the mRNAs. It was reported previously that lncRNAs in the brain are less conserved than mRNAs in different regions, and that the level of conservation among different species is low [42,43]. Additionally, the low conservation of lncRNAs was verified in goat muscle with other species (cow, human, and mouse), but the conservation was higher between goat and human than cow and mouse.

Temporal-specific expression patterns reveal the functions of lncRNAs within muscle at specific times during development. Several studies have already revealed that lncRNAs tend to show stage-specific expression [38,44]. With the early module, the enriched GO terms indicated that the target genes were related to muscle fiber structure, thereby revealing the formation of muscle fibers from F45 to F90. It was found that the target genes of lncRNAs from the M1 module participate in muscle regeneration, structural formation, and muscle fiber types. For example, Troponin T (*TNNT*) is a central player in the calcium regulation of actin thin filament function and is essential for the contraction of striated muscles [20]. Our results indicated that *TNNT1* is related to LNC_010184 and LNC_010185, *TNNT2* is related to LNC_009262 and LNC_009263, and that there are type-specific *TNNT* isoforms in slow skeletal muscle and cardiac muscle [20]. However, the hub- LNC_011371 of M2 targets 74 genes (such as *MB, CLIC5*, and others) and they show up-regulation after birth. The growth of skeletal muscle in the later stages is mainly due to the hypertrophy of muscle fibers, and inadequate oxygen supply or hypoxia limits hypertrophy [13,45]. As an essential member of muscle fibers, MB can maintain maximal 286 oxygen uptake (VO2 max) during muscle fiber hypertrophy by transporting oxygen [45]. Our functional analysis suggested that the late lncRNAs were related to metabolism, muscle differentiation, and hypertrophy genes. *AXIN1*, which is related to LNC_012926, is a hypomethylated gene that shows enhanced expression after loading and maintains its hypomethylated status even during unloading where muscle mass return to control levels, indicating a memory of *AXIN1* methylation signatures following earlier hypertrophy [46]. The transcription coactivator *Eya2*, which was found previously to be up-regulated during physiological hypertrophy, activates the expression of mTOR which is a critical mediator of physiological hypertrophy [28]. Notably, some of hub-LNC_007561 targets genes directly regulate muscle fiber development, maintain muscle stability and development such as *SDC3*, *MYO10*, and *TCF4* [27,47,48]. For the lncRNAs in M1, LNC_006306 and LNC_003007 are generated from the *APC* which is required for muscle stem cell proliferation [18]. Interestingly, hub-LNC_001728 binds to S100 A4 and has a consistent trend with it in all stages. The S100 A4 was up-regulated in F90 to F120, and there are reports that the up-regulation of S100 A4 promotes cardiomyocyte production and increases myocardial cell number by inhibiting apoptosis. Therefore, we presume that S100 A4 might involve in myocyte proliferation and production between F90 to F120 by binding to LNC_001728 [49]. Moreover, 6 of 22 lncRNAs in M5 are produced from the host gene *RTL1*, which is important for postpartum skeletal muscle development [50].

## 4. Materials and Methods

### 4.1. Animal Care and Tissue Collection

Anhui white goats (AWG) were raised on the farm of Boda Company (Hefei) under a unified field management system. The female AWG goats were immersed in CIDR for 12 days for estrus at the same time, and artificial insemination techniques were used for the ewes who had succeeded in estrus. Twenty-one goats that had been mated successfully were selected for feeding, and the fetuses and kids muscles of different periods were taken as samples. Cesarean sections were conducted at 45 (F45), 65 (F65), 90 (F90), 120 (F120), and 135 (F135) days to obtain the fetuses. The kids born within 24 h (B1) and 90 days (B90) were also collected. Three biological replicates were included for each developmental stage and the longissimus dorsi muscles were dissected from the obtained fetuses and kids. The 21 samples in total were immediately frozen in liquid nitrogen until needed for RNA extraction. This study was carried out in accordance with the principles of the Basel Declaration and recommendations of the Guide for the Care and Use of Laboratory Animals (http://grants1.nih.gov/grants/olaw/references/phspol.htm). The protocol was approved by the ethics committee of Anhui Agricultural University under permit No.AHAU-AE2017-07 (May 12 2017).

### 4.2. RNA Isolation, Lncrna and Small RNA Library Preparation and Sequencin

Total RNA was isolated from the 21 samples using TRIzol reagent (Invitrogen, Carlsbad, CA, USA). RNA degradation, contamination and purity were detected using 1% agarose gels and a NanoPhotometer^®^ (IMPLEN, Los Angeles, CA, USA). RNA concentrations were measured using a Qubit^®^ RNA Assay Kit and Qubit^®^ 2.0 Fluorometer (Life Technologies, Carlsbad, CA, USA). The RNA integrity was assessed using the RNA Nano 6000 Assay Kit for the Bioanalyzer 2100 system (Agilent Technologies, Santa Clara, CA, USA).

A total of 3 μg RNA per sample was used as the input material for RNA purification. Firstly, ribosomal RNA was removed using an Epicentre Ribo-zero™ ethanol precipitation. Sequencing libraries were subsequently generated using the rRNA depleted RNA with NEBNext^®^ Ultra™ Directional RNA Library Prep Kit for Illumina^®^ (NEB, Ipswich, MA, USA) under the manufacturer’s recommendations. Next, 3 μL of USER Enzyme (NEB, Ipswich, MA, USA) was used with size-selected, adaptor-ligated cDNA, and PCR was performed with Phusion High-Fidelity DNA polymerase, Universal PCR primers and Index (X) Primer (Generay Biotech Co. Ltd., Shanghai, China). Finally, products were purified (AMPure XP system) and library quality was assessed on the Agilent Bioanalyzer 2100 system. The clustering of index-coded samples was performed using the cBot Cluster Generation System using TruSeq PE Cluster Kit v3-cBot-HS (Illumia). After cluster generation, the libraries were sequenced on an Illumina Hiseq 4000 platform and 150 bp paired-end reads were generated.

### 4.3. Data Analysis

In this step, clean reads were obtained by removing reads containing adapter, reads containing ploy-N, and low-quality reads from the raw data. At the same time, the Q20, Q30, and GC content of the clean data were calculated. All of the downstream analyses were based on clean and high-quality data. The *Capra hircus* reference genome and gene model annotation files were downloaded from the genomics website (https://www.ncbi.nlm.nih.gov/genome/?term=goat). The index of the reference genome was built using Bowtie v2.2.8 and paired-end clean reads were aligned to the reference genome using HISAT2 v2.0.4 [17,51]. The mapped reads of each sample were assembled using StringTie (v1.3.1) via a reference-based approach. Transcripts with coding potential were filtered by CNCI, CPC, and Pfam-scan, and the non-coding transcripts were selected as our candidate lncRNA collection [52,53,54].

### 4.4. Conservation Analysis

Cow, human, and mouse lncRNA sequences were downloaded from the NONCODE database (http://www.noncode.org/download.php) and compared with the lncRNAs in our datasets by BLAST. Conservation scoring of human known lncRNAs was analyzed using phastCons software and lncRNA sequences with score above 0.8 were selected and sequence aligned by two-way BLAST in our datasets.

### 4.5. Quantification of Gene Expression Level and Differential Expression Analysis

Cuffdiff (v2.1.1) was used to calculate FPKMs of both lncRNAs and coding genes in each sample [55]. FPKM denotes fragments per kilo-base of exon per million fragments mapped and is calculated based on the length of the fragments and reads count mapped to this fragment. Gene FPKMs were computed by summing the FPKMs of transcripts in each gene group. The Ballgown suite includes functions for interactive exploration of the transcriptome assembly, visualization of transcript structures, and feature-specific abundances for each locus, and post-hoc annotation of assembled features to annotated features. Transcripts with a *p*-value of 0.05 were assigned as differentially expressed.

### 4.6. Quantitative RT-PCR Validation

Six differentially expressed lncRNAs were randomly selected for quantitative RT-PCR (QRT-PCR) using GoTaq qPCR Master Mix (Promega, Madison, WI, USA) and Real-time Thermal Cycler 5100 (Thermo, Waltham, MA, USA). All primer pairs (Table S9) used for PCR amplification were designed using NCBI and synthesized by the Shanghai Generay. Biotech Co. Ltd. The housekeeping gene, β-actin (ACTB), was amplified as a control. The amount of target sequence normalized to the reference sequence was calculated as 2^−ΔΔCt^. Statistical analysis was performed on normalized data using SPSS 21.0 for Windows. Data are presented as means ± SEM and were considered statistically significant at *p*-value < 0.05.

### 4.7. Target Gene Prediction

A previous study has shown that lncRNAs exert cis-regulatory effects on their co-localized genes [56]. Co-location role is lncRNA acting on neighboring target genes. The 100 k coding genes upstream and downstream of lncRNA were searched to identify their cis-acting effects. Additionally, The co-expression role is that lncRNA recognizes targeted genes by expression level. Pearson correlation coefficient method was used to analyze the correlation between lncRNA and mRNA, and the mRNA with the absolute correlation value greater than 0.95 was used for functional enrichment analysis to predict the main function of lncRNA.

### 4.8. Functional and Pathway Enrichment Analysis

Gene Ontology (GO) is a classification system for internationally standardized gene functions that provides a controlled vocabulary to describe the properties of genes and gene products comprehensively. GO enrichment analysis of differentially expressed genes or lncRNA target genes were implemented using the GOseq R package, in which gene length bias was corrected. Kyoto Encyclopedia of Genes and Genomes (KEGG) is a database resource for understanding high-level functions and utilities of the biological system. We used KOBAS software to test the statistical enrichment of differential expression genes or lncRNA target genes in KEGG pathways.

### 4.9. WGCNA Network Analysis

The genes which were differentially expressed between all stages of goat skeletal muscle development were selected using R package WGCNA [57]. A signed weighted correlation network was constructed by first creating a matrix of Pearson correlation coefficients between all pairs of genes across the measured samples. The adjacency matrix was then transformed into a topological overlap matrix (TOM) to minimize the effects of noise and spurious associations. To define modules as branches, we employed the Dynamic Tree Cut algorithm with default parameters to cut the hierarchal clustering tree [58].

## 5. Conclusions

We described for the first time a seven-stage lncRNA expression profile in goat skeletal muscle development by RNA-seq and characterized the temporal patterns of DElncRNAs. Due to its specific expression module, we speculated that the developmental stage from F45 to F90 may be the most critical period for the regulation of muscle structural formation and the establishment of muscle fiber types. The F135 to B90 stage may involve the regulation of muscle hypertrophy and metabolism. Moreover, the biggest difference in the lncRNA expression profiles was found at F120, confirming that the transition from early to late development occurs at this period. We will validate these findings in future studies to gain a deeper understanding of the functional roles of the lncRNAs in mammalian muscle development.

## Figures and Tables

**Table 1:** Five muscle-related targeting mRNAs of goat skeletal muscle LncRNAs in each module.

mRNAs	Transcript Source	Function of mRNA	Associated lncRNAs
APC	Module1	APC is required for muscle stem cell proliferation and skeletal muscle tissue repair [18].	LNC_006306; LNC_003007
SP1	Module1	Sp1 is an activator of MyoD and a suppressor of CDKN1 A that plays an important role in bovine muscle cell proliferation and differentiation [19].	LNC_002771; LNC_002368; LNC_009507; LNC_011751
TNNT2	Module1	TNNT2 is a muscle type-specific TNT for cardiac muscle [20].	LNC_009262; LNC_009263
MYO1 C	Module1	Myo1 c regulates glucose uptake in mouse skeletal muscle [21].	LNC_010262; LNC_010263
MYF5	Module1	Myf5 in muscle regeneration indicates that their expression after injury stabilizes myogenic identity and confers the capacity for muscle differentiation [22].	LNC_003108; LNC_003109; LNC_003110; LNC_003111; LNC_003112; LNC_003113;
JAK3	Module1,2	JAK3 inhibition potently facilitates myoblast differentiation through antagonistic STAT1/STAT3 activities [23].	LNC_003114
MEF2 A	Module2	Requirement of MEF2 A, C, and D for skeletal muscle regeneration and differentiate [24].	LNC_004659; LNC_004660
MYH13	Module2,5	MYH13 associates with extraocular muscles [25].	LNC_010326; LNC_010329
TNNT1	Module1,2	TNNT1 is a muscle type-specific TNT for slow skeletal muscle [20].	LNC_010184; LNC_010185
MYOT	Module3	Mutation in the myotilin gene (MYOT) causes a severe form of limb girdle muscular dystrophy 1 A [26].	LNC_004077
TCF4	Module3	TCF4 regulates myogenesis [27].	LNC_000994; LNC_006624; LNC_007561
EYA2	Module3	Eya2 is a critical regulator of physiological hypertrophy [28].	LNC_000994; LNC_001379; LNC_006624
MEOX1	Module3	Meox1 initiates G2 cell-cycle arrest within muscle stem cells [29].	LNC_004123
BAMBI	Module3	BAMBI promotes C2 C12 myogenic differentiation by enhancing Wnt/β-catenin signaling [29].	LNC_014703
MYO10	Module3	Myosin is a component of myofibrils [30]	LNC_005183; LNC_007561; LNC_013466
MARK2	Module4	Mark2 (also known as Par1 b) is an important regulator of cell polarity [31].	LNC_014128
MEF2 C	Module4	Requirement of MEF2 A, C, and D for skeletal muscle regeneration and differentiation [24].	LNC_004357
MYC	Module4	Myc is involved in regulating myoblast proliferation during muscle development and regeneration [32].	LNC_008001
BRCA1	Module4	BRCA1 is a regulator of metabolic function in skeletal muscle [33]	LNC_010762; LNC_010763; LNC_010766
TNNT3	Module4	TNNT1 is a muscle type-specific TnT for fast skeletal muscle [20].	LNC_014032
LOC102180883 (myosin-4)	Module5	Myosin is a component of myofibrils [30].	LNC_010329
LOC102181155 (myosin-2)	Module5	Myosin II is the myosin type responsible for producing muscle contraction in muscle cells in most animal cell types [34].	LNC_010329
LOC102181426 (myosin-1)	Module5	Myosin is a component of myofibrils [30].	LNC_010329
MYH8	Module5	MYH8 is associated with developing muscle [25].	LNC_010329
RTL1	Module5	Ectopic expression of PEG11/RTL1 contributes to the callipyge muscular hypertrophy [35].	LNC_010329

## Data Availability

Data in the manuscript was unpublished. The datasets analyzed during the current study are available from the corresponding author on reasonable request. If the article is accepted for publication, the data availability statement will be published as part of the accepted article.

## Supplementary Materials

Supplementary materials can be found at https://www.mdpi.com/1422-0067/20/16/3950/s1. Figure S1. Heat map of differentially expressed genes. (**a**,**b**) Hierarchical clustering heat map of differentially expressed lncRNAs and miRNAs by samples. Red: relatively high expression; Blue: relatively low expression. Figure S2. Main lncRNA-mRNA subnetwork of module 2. (**a**) The red diamond represents lncRNA; yellow circle represents co-location mRNAs, blue circle represents co-expression mRNAs 714, and the size is expressed in degrees. Figure S3. Main lncRNA–mRNA subnetwork of module 3. (**a**) The red diamond represents lncRNA, yellow circle represents co-location mRNAs, blue circle represents co-expression mRNAs, and the size is expressed in degrees. Figure S4. Main lncRNA–mRNA subnetwork of module 1. (**a**) The red diamond represents lncRNA, yellow circle represents co-location mRNAs, blue circle represents co-expression mRNAs, and the size is expressed in degrees. Table S1. The summary information of transcriptome. Table S2. The detected miRNAs, lncRNAs, and mRNAs in all the libraries. Table S3. LncRNAs with a conservation score > 0.8 in the human transcriptome. Table S4. Two-way BLSAT of goat skeletal muscle LncRNAs with human LncRNAs that have a conservation score > 0.8. Table S5. Differently expressed mRNAs during the seven stages. Table S6. Differently expressed lncRNAs during the seven stages. Table S7. The GO terms enrichment of lncRNA modules. Table S8. The KEGG enrichment of lncRNA modules. Table S9. Primer pairs used for qRT-PCR amplification.

## Abbreviations

LncRNA	Long non-coding RNA
F45	Fetus 45 days
F65	Fetus 65 days
F90	Fetus 90 days
F120	Fetus 120 days
F135	Fetus 135 days
B1	Born 1 day
B90	Born 90 days
AWG	Anhui white goats
FPKM	Fragment Per Kilobase of exon per Million fragments mapped
WGCNA	Weighted gene co-expression network analysis
PCA	Component analysis
DElncRNAs	Differentially expressed lncRNAs
DEmRNAs	Differentially expressed mRNAs
