# Supplementary figures and images for: Comprehensive Analysis of LncRNA Reveals the Temporal-Specific Module of Goat Skeletal Muscle Development
Source: Int J Mol Sci. 2019 Aug 14;20(16):3950. doi: 10.3390/ijms20163950 (PMC6719106; doi:10.3390/ijms20163950)

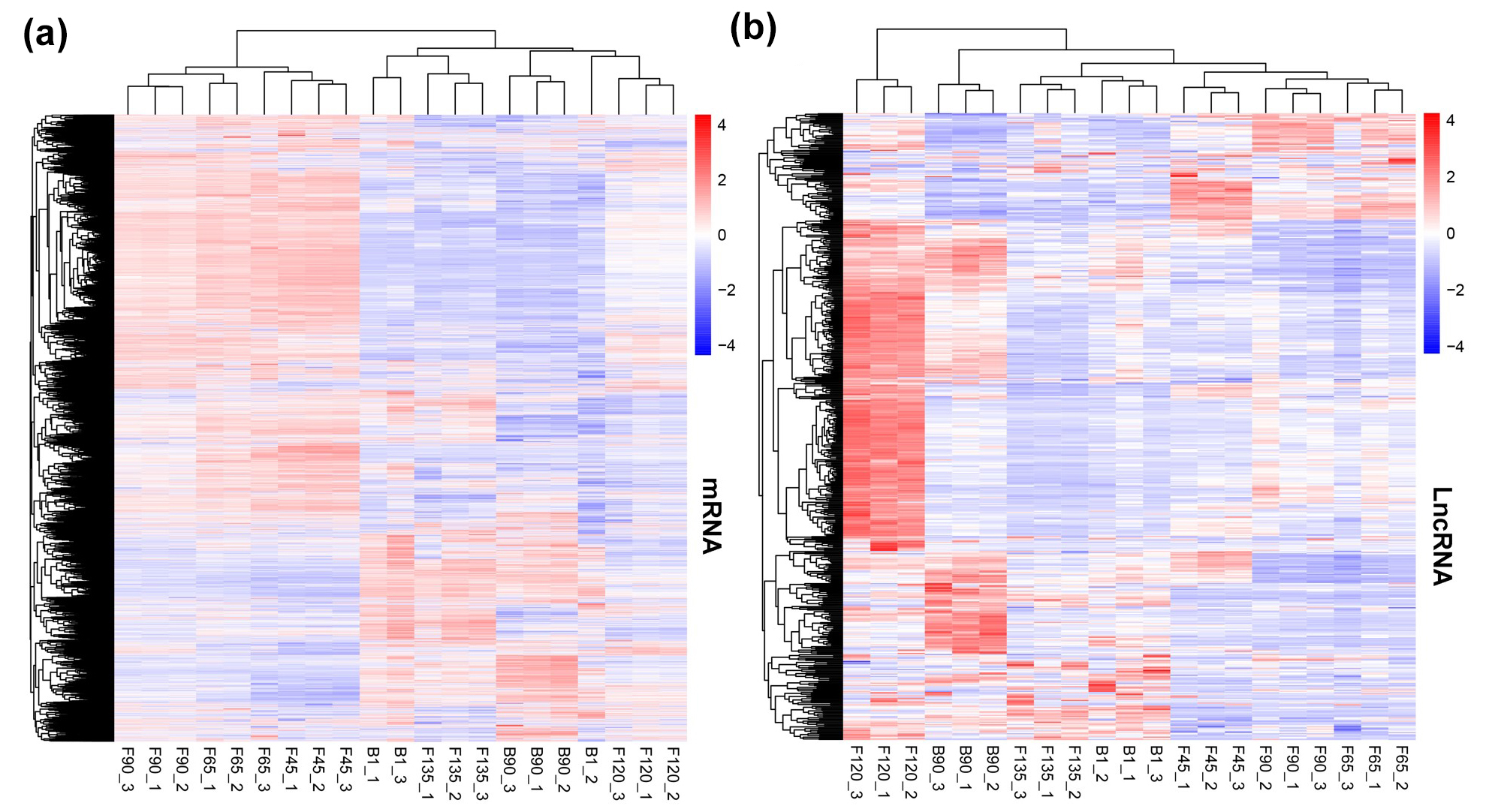

Supplement: Supplementary file 1 [file ijms-20-03950-s001.zip › supplyments/Fig.sup 1.jpg]

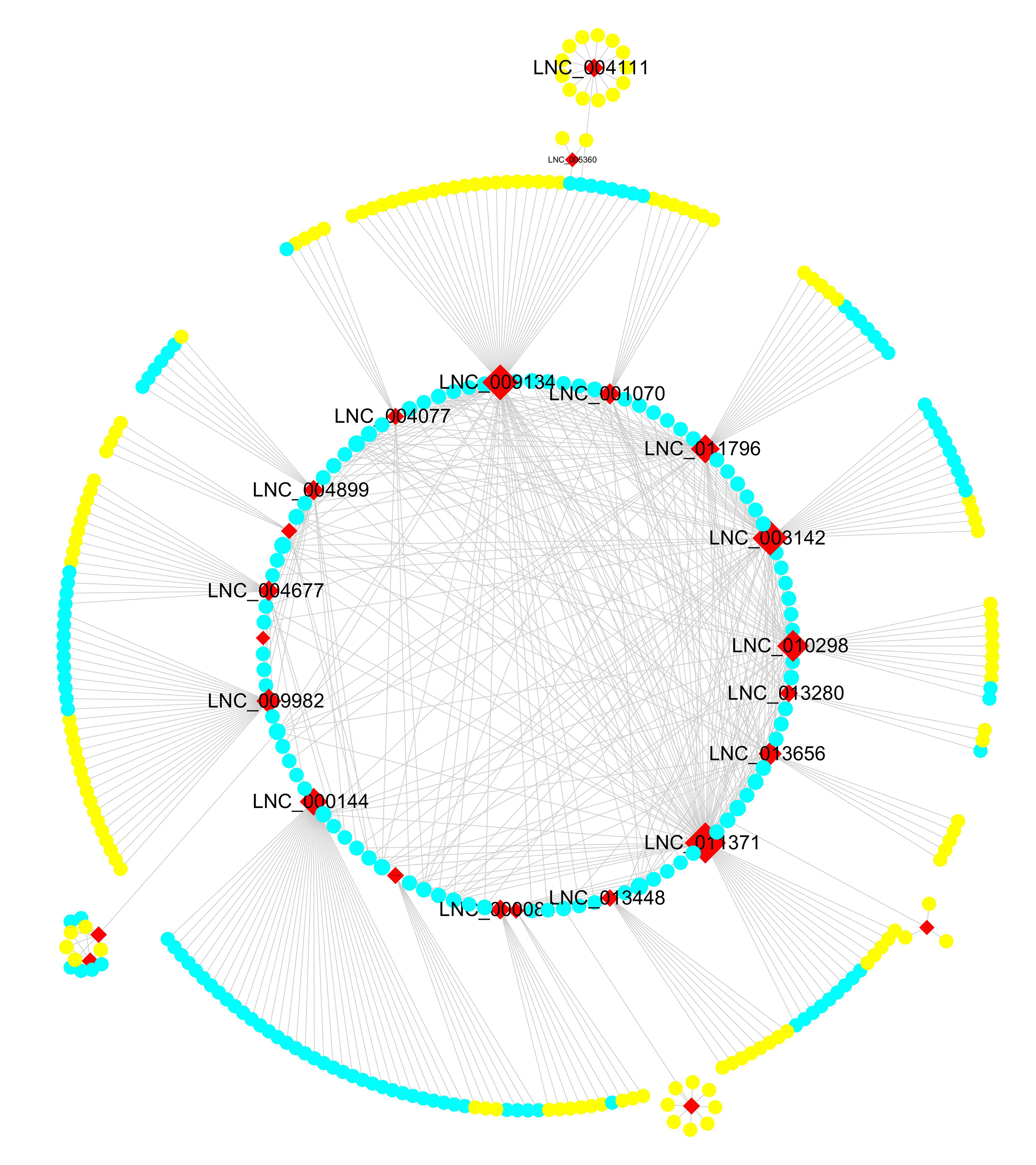

Supplement: Supplementary file 1 [file ijms-20-03950-s001.zip › supplyments/Fig.sup2.jpeg]

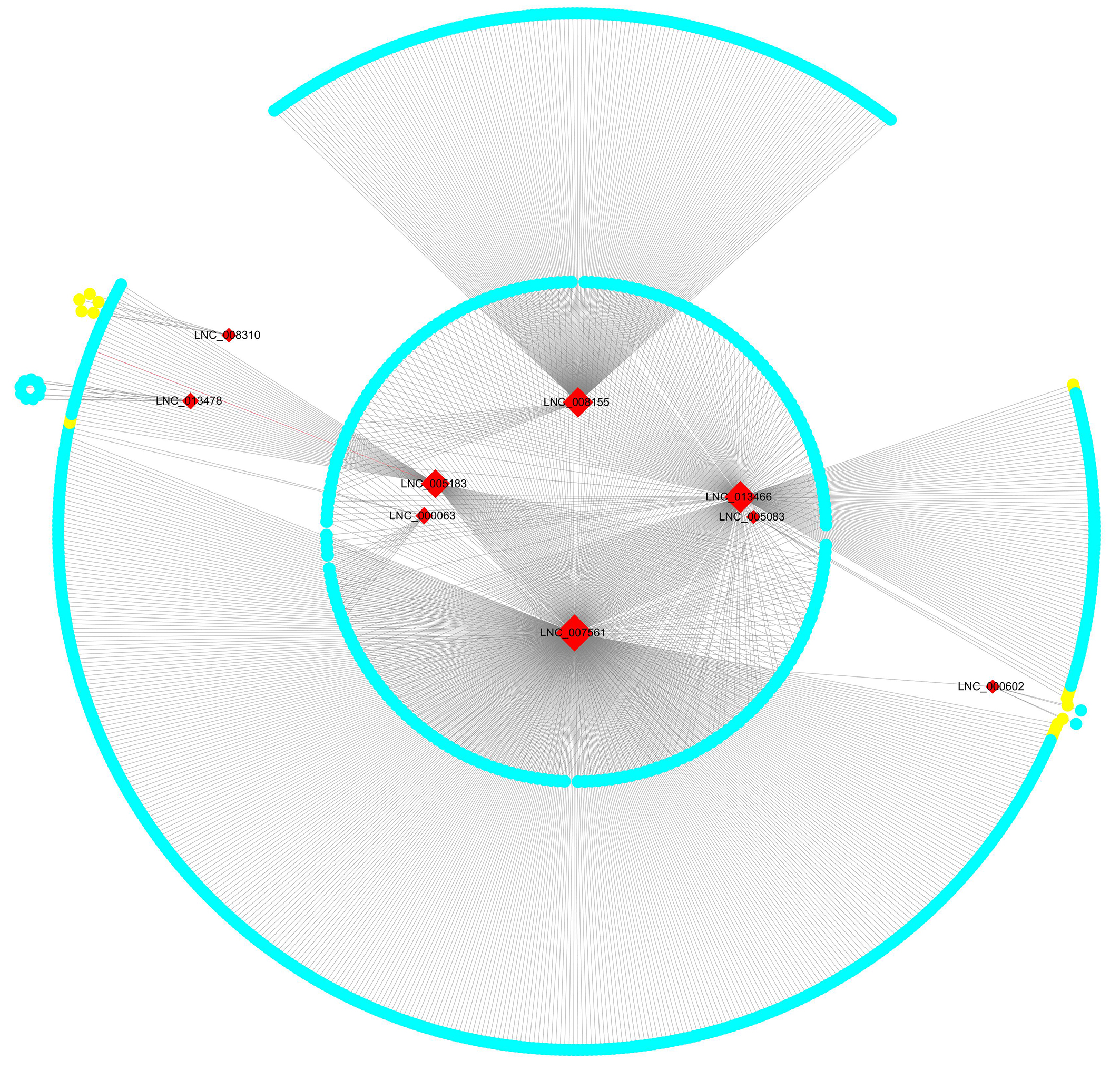

Supplement: Supplementary file 1 [file ijms-20-03950-s001.zip › supplyments/Fig.sup3.jpg]

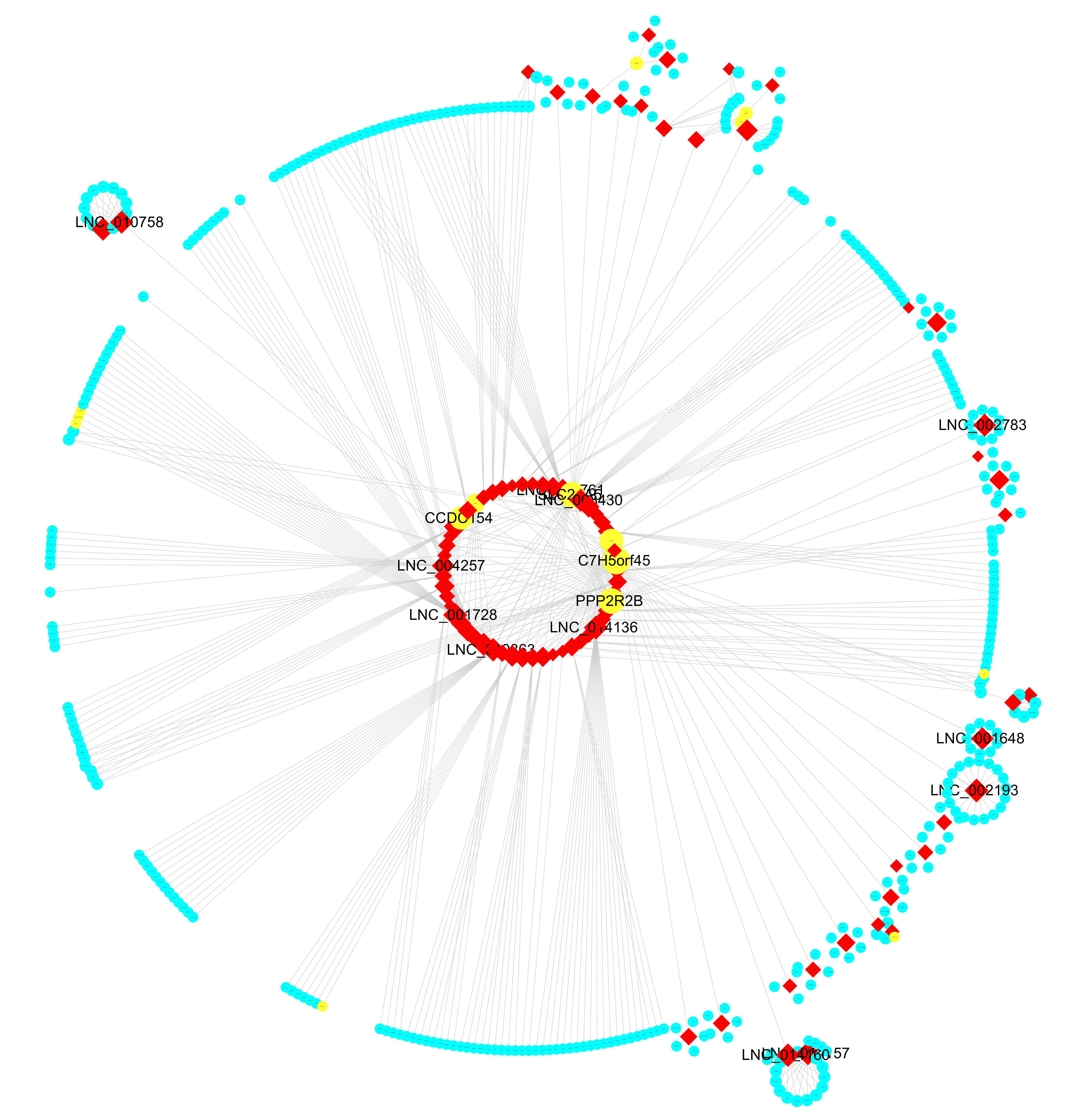

Supplement: Supplementary file 1 [file ijms-20-03950-s001.zip › supplyments/Fig.sup4.jpg]
